# Yes, we CAM! First evidence of CAM photosynthesis in a carnivorous plant

**DOI:** 10.1111/plb.70128

**Published:** 2025-11-10

**Authors:** N. J. Fleck, T. F. E. Messerschmid, A. Fleischmann, R. C. Ferrari, G. Kadereit

**Affiliations:** ^1^ Biocenter, Ludwig Maximilian University of Munich Martinsried Germany; ^2^ Botanischer Garten München‐Nymphenburg (SNSB‐BGM) Munich Germany; ^3^ Botanische Staatssammlung München (SNSB‐BSM) Munich Germany; ^4^ GeoBio‐Center LMU, Ludwig Maximilian University of Munich Munich Germany; ^5^ Prinzessin Therese von Bayern Lehrstuhl für Systematik, Biodiversität und Evolution der Pflanzen Munich Germany

**Keywords:** Carnivorous plants, crassulacean acid metabolism, facultative CAM, heterophylly, *Pinguicula*, titratable acidity

## Abstract

- Crassulacean acid metabolism (CAM) is a photosynthetic adaptation enabling higher CO_2_ concentration close to Rubisco and increased water use efficiency. It evolved in at least 38 plant families, none reported to be carnivorous. Here, we investigate CAM activity in the carnivorous genus *Pinguicula*, so far reported as C_3_, with succulent leaves and distributed mainly in Central America.
- Eight species of *Pinguicula*, most with seasonal heterophylly, were cultivated under controlled conditions and monitored for changes in diel acidification (ΔH^+^) when grown with abundant or limited water availability. Additionally, leaf anatomy and gas exchange were studied in representative species.
- In the winter trial, five species had positive and significant ΔH^+^ 1 week after withholding water (*P. agnata, P. esseriana, P. gigantea, P. laxifolia* and *P. moranensis*). ΔH^+^ levels were in the range previously reported in weak, facultative CAM plants (15–30 μmol H^+^ g^−1^ FW). The summer trial revealed positive ΔH^+^ for most species tested, regardless of water availability. Three of the homophyllous species had an unclear pattern of CAM induction (*P. emarginata, P. martinezii*) or no CAM induction (*P. grandiflora*). Gas exchange in *P. agnata* found no dark CO_2_ assimilation, suggesting CAM cycling.
- We present the first report of CAM in a carnivorous plant, reinforcing the need to search for CAM in other families. Future work should assess reversibility of the C_3_‐CAM transition, explore the interplay between nutrient and carbon balance, and the contribution of weak CAM to plant fitness.

## Graphical abstract

Evidence for weak, facultative CAM is reported for the first time in a carnivorous plant, i.e., in Mexican representatives of *Pinguicula* (Lentibulariaceae).

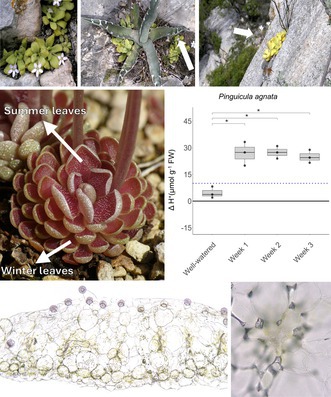

## INTRODUCTION

Since the first published observations of crassulacean acid metabolism in the 19th century (de Saussure 1804), this specialized photosynthesis pathway has been detected in 370 genera from 38 families, with at least 66 independent evolutionary origins, and is estimated to occur in about 7% of all vascular plant species (Gilman *et al*. 2023). Considering its flexibility and cryptic nature compared to C_4_ photosynthesis, it is likely that many more origins of CAM are yet to be found. CAM typically involves an ecological strategy to reduce water loss. During the night, CAM plants take up CO_2_ and convert it into malic acid, which is stored in vacuoles of mesophyll cells. During daytime, this acid is remobilized and decarboxylated to supply CO_2_ to Rubisco, allowing stomata to remain closed (Osmond 1978). This set of features results in a marked circadian acidification rhythm that is exclusive to CAM plants.

Different types of CAM have been defined according to its contribution to total carbon uptake and inducibility (Winter 2019). CAM plants, for example, cacti and agaves, acquire most of their carbon via nocturnal CO_2_ assimilation, with strong CAM defined as dark CO_2_ fixation rates of ca. 5 μmol m^−2^ s^−1^ and nocturnal acidification (ΔH^+^) of ca. 200 μmol H^+^ g^−1^ FW (Winter *et al*. 2011; Winter 2019). In these plants, CAM expression is obligate. If CAM is expressed optionally or reversibly and triggered by environmental cues, such as drought, it is referred to as facultative CAM. Facultative CAM plants still obtain most of their carbon via the C_3_ cycle, with nocturnal ΔH^+^ as low as 3 μmol H^+^ g^−1^ FW (Winter *et al*. 2018, 2021). Other inconspicuous forms of weak CAM include CAM cycling and CAM idling. In CAM cycling, gas exchange follows a C_3_‐like cycle, with CO_2_ uptake only during daytime, but ΔH^+^ derived from nocturnal fixation of respiratory CO_2_. CAM idling, on the other hand, is found in severely stressed plants, when there is no net CO_2_ uptake and ΔH^+^ is also significant (Winter 2019).

The most reliable method for detecting CAM are titratable acidity assays, which can resolve day–night variations as small as 1–2 μmol H^+^ g^−1^ FW (Winter 2019; Winter & Smith 2021). Another indicator for CAM used to be the analysis of stable carbon isotope ratios (δ^13^C) (Bender *et al*. 1973; Osmond *et al*. 1973). Whilst this is useful tool to identify constitutive, strong CAM in broad surveys, it is not suitable for detecting facultative, weak CAM. Lineages displaying photosynthetic flexibility might present values oscillating around −20‰, which is the threshold for distinguishing between CAM and C_3_, and thus do not provide conclusive evidence of CAM (Winter and Holtum 2002; Winter 2019; Messerschmid *et al*. 2021). Not only can δ^13^C values vary according to environmental conditions, but most of the carbon pool also originates from C_3_ metabolism in weak CAM, with values more negative and C_3_‐like (Winter 2019; Messerschmid *et al*. 2021; Winter & Smith 2021; Gilman *et al*. 2023). Even plants acquiring most of their carbon via CAM (i.e. strong CAM) sometimes have C_3_‐like δ^13^C values (Winter and Holtum 2002).

**Fig. 1:**
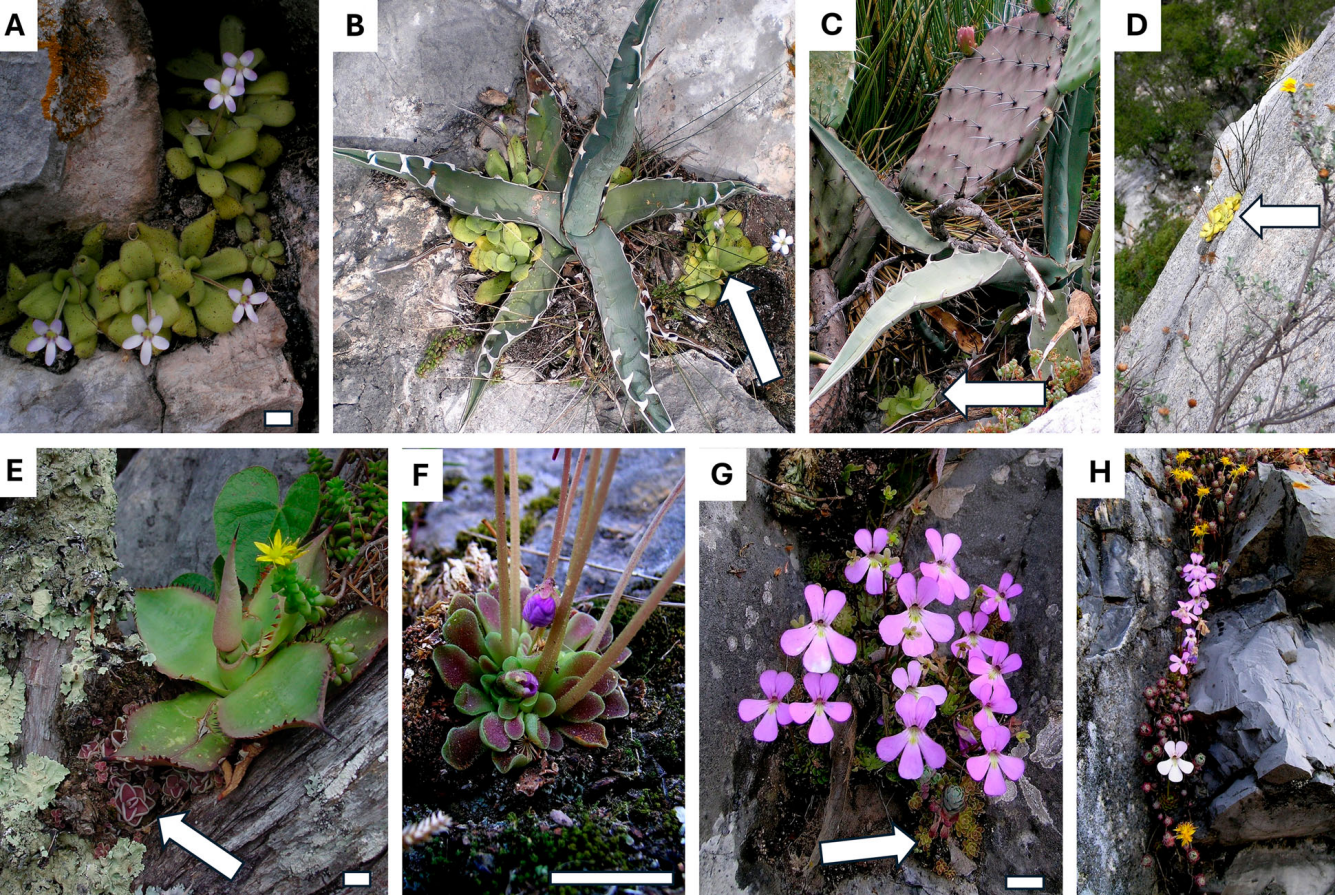
Habitat, habit and morphology of Mexican dry‐growing *Pinguicula*. (A–D) *P. agnata*. E *P. esseriana*, summer leaves. (F–H) *P. esseriana*, winter leaves and flowers. In these habitats, plants frequently co‐occur with other CAM plants, for example, *Agave* sp. (Asparagaceae; B, C, E), *Sedum* sp. (Crassulaceae; C, E, G, H), *Opuntia* sp. (Cactaceae; C). White arrows indicate *Pinguicula* individuals. Scale bars = 1 cm. Photos taken by Fernando Rivadavia at Río Moctezuma Canyon, Hidalgo state (A–D), El Tepozán, Querétaro state (E), and a pass near Ciudad Victoria, Tamaulipas state (F–H).

Less is known about environmental triggers of CAM other than drought, but nutrient availability has been reported to play a role (Pereira & Cushman 2019). One strategy to survive in nutrient‐poor habitats is carnivory. More than 860 carnivorous plant species are known, which evolved independently in at least five angiosperm orders: Caryophyllales, Oxalidales, Ericales, Lamiales and Poales (Fleischmann *et al*. 2018). To our knowledge, all studied carnivorous plants so far perform C_3_ photosynthesis, with low (<3 μmol m^−2^ s^−1^) net CO_2_ assimilation (*A*) rates (Pavlovič 2022), even though there is a note on the C_4_‐like leaf anatomy of a Mexican species of *Pinguicula* by Studnička (1991). *Pinguicula* (Lentibulariaceae) comprises ca. 115 species of rosetted, herbaceous carnivorous plants, with more than half found in Central America (Roccia *et al*. 2016; Fleischmann & Roccia 2018; Fleischmann 2021; Shimai *et al*. 2021; López‐Pérez *et al*. 2024). Based on their distribution and growth pattern, three morphological groups are distinguished (Casper 1966; Roccia *et al*. 2016; Fleischmann & Roccia 2018): (i) temperate species with heterophyllous growth that produce carnivorous leaves in summer and a compact resting leaf bud as a hibernaculum in winter; (ii) tropical species with heterophyllous growth, with carnivorous leaves in the wet season and a dense rosette of smaller, more succulent non‐carnivorous leaves in the dry season (Fig. 1); (iii) homophyllous species that produce a single carnivorous leaf type and are either annuals or perennials in climatically stable habitats. In carnivorous leaves, the leaf adaxial side is covered with two types of secretory glands: stalked glands secreting small droplets of sticky, aqueous muco‐polysaccharides to trap prey, and sessile glands to produce digestive enzymes and take up nutrients (Heslop‐Harrison & Knox 1971; Heslop‐Harrison & Heslop‐Harrison 1981; Vassilyev & Muravnik 1988). Rarely, the abaxial side also bears glands, as in *P. gigantea*.

Overall, the interplay between C assimilation vs nutrient and water intake in Mexican *Pinguicula* species is particularly interesting, when considering their distribution in seasonally dry environments. Succulence has mainly been reported in winter leaves, leading to the suggestion that CAM might occur in this genus (Studnička 1991; Pavlovič 2022), although no evidence has yet been presented (Adamec *et al*. 2021). Interestingly, δ^13^C values in *P. agnata* and *P. gigantea* between −14‰ and −12‰ were reported by Lin *et al*. (2025). However, this same work also reported δ^13^C values of ca. −15‰ for *Beta vulgaris* and *Ocimum basilicum*, two C_3_ plants with expected δ^13^C values of −25 to −30‰.

In the current study, seven Mexican and one European species of *Pinguicula* were cultivated under controlled conditions and subjected to 3 weeks of water withholding treatment to generate stressful conditions. Two experiments were performed, a winter and a summer trial, considering heterophylly in some species. Monitoring circadian fluctuations in titratable acidity for these species revealed a significant increase in ΔH^+^ as water became scarce during the winter trial, but this pattern was not repeated in the summer trial. Although no net dark CO_2_ assimilation was observed in a gas exchange experiment, a reduction in respiratory CO_2_ emission at night in *P. agnata* is consistent with the induction of CAM cycling following withholding of water. We document the first conclusive evidence of CAM photosynthesis in carnivorous plants, suggesting more widespread occurrence of CAM across plant families.

## MATERIAL AND METHODS

### Plant material and growth conditions

Plant material was obtained in December 2022 for the winter trial, and June 2023 for the summer trial. Four species were available at the Botanic Garden Munich‐Nymphenburg (*P. agnata*, *P. emarginata*, *P. gigantea* and *P. moranensis*), while the remaining species were purchased from a specialized commercial provider (Gartenbau Thomas Carow, Nüdlingen, Bayern, Germany). A list of the studied species, together with original distribution, habitat, and leaf description is presented in Table 1. Voucher information is given in Table S1. In general, the natural habitat of these species is north‐facing cliffs, on which they grow in spaces between rocks, often beneath the vegetation. Hence, they are usually not exposed to full sunlight in the wild (Lampard *et al*. 2016).

**Table 1:** List of studied *Pinguicula* species.

Species	Distribution	Habitat	Leaf description	References	Winter/summer trials
*Pinguicula agnata* Casper	NE Mexico	Subtropical, perennial, tropical deciduous forests or submontane mattoral shrubland at 350–2000 m	*Heterophyllous*: strongly succulent, non‐carnivorous winter leaves (7.5–30 mm) with non‐glandular hairs versus larger, succulent carnivorous summer leaves (35–80 mm)	Lampard *et al*. 2016	+/+
*Pinguicula emarginata* Zamudio Ruiz and Rzedowski	SE Mexico, Veracruz, Puebla	Subtropical cloud forest with high humidity, shady, rocky habitats, perennial, hibernaculum absent	*Homophyllous*: leaves succulent, carnivorous leaves (10–50 mm) throughout the year	Lampard *et al*. 2016	+/−
*Pinguicula esseriana* Kirchner ‘El Huizache’ (San Luis Potosí state ‐ type locality)	CE Mexico, San Luis Potosí to Tamaulipas	Subtropical, perennial, montane, hot semi‐arid steppe habitats	*Heterophyllous*: small, strongly succulent, non‐carnivorous winter leaves (up to 8 mm) sparse non‐glandular hairs versus larger, non‐succulent, carnivorous summer leaves (up to 36 mm)	Kirchner 1981; Lampard *et al*. 2016	+/+
*Pinguicula esseriana* ‘Ciudad Victoria’ (Tamaulipas state)	CE Mexico, near Ciudad Victoria, Tamaulipas	Subtropical, perennial, in vertical fissures of feldspar in xerophytic shrub vegetation	*Heterophyllous*: morphology like *P. esseriana* from the type locality	Lampard *et al*. 2016	+/−
*Pinguicula gigantea* Luhrs	SW Mexico, Oaxaca	Subtropical, perennial, on steep slopes, winter and summer leaves ± uniform, hibernaculum absent	*Homophyllous*: leaves strongly succulent, comparatively large (60–145 mm) and densely glandular on both sides	Luhrs 1995	+/−
*Pinguicula grandiflora* Lam.	W Europe	Temperate, moist, shady habitats on calcareous soils	*Heterophyllous*: winter rosette with small leaves, spring and summer leaves ± uniform and carnivorous (30–60 mm)	Roccia *et al*. 2016	−/+
*Pinguicula laxifolia* Luhrs	CE Mexico, only known from Gomez Farias District, Tamaulipas	Subtropical, perennial, 1900–2070 m in more arid region beyond cloud forest zone on shaded, almost vertical limestone outcrops	*Heterophyllous*: winter leaves (10–17 mm) slightly succulent, non‐carnivorous, densely non‐glandular hairs versus much bigger, slender, carnivorous summer leaves (40–68 mm)	Lampard *et al*. 2016	+/+
*Pinguicula martinezii* Zamudio	CE Mexico, plains of Chiquito, Querétaro	Subtropical, perennial, montane mesophytic forests at 2000–2370 m on almost vertical limestone escarpments	*Homophyllous*: winter and summer leaves ± uniform in shape but differ in size 8–30 mm and 50–100 mm, respectively, slightly succulent, both carnivorous	Lampard *et al*. 2016	+/+
*Pinguicula moranensis* Kunth	N Mexico to Guatemala and N El Salvador	Subtropical, perennial, high habitat diversity, from hot and humid tropical deciduous forests to cooler, montane forests, at 100–3200 m from −5°C to 35°C	*Heterophyllous*: small, succulent winter leaves (10–30 mm) with non‐glandular hairs versus much larger, thin, nearly translucent, carnivorous summer leaves (60–115 mm)	Lampard *et al*. 2016	+/+

Experiments were conducted on nine accessions, comprising eight species of *Pinguicula*, all Mexican species from the monophyletic lineage of *Pinguicula* subgenus *Temnoceras*, except *P. grandiflora* which belongs to subgenus *Pinguicula* and is distributed in W. & SW. Ireland to NW. Spain and the Swiss Jura (for accession numbers see Table S1).

For the current work, plants were grown in pots with a peat‐based substrate according to their size and regularly fertilized with commercially available products. For each species, three individuals corresponding to vegetative clones of comparable size were acclimated for at least 1 week in a greenhouse simulating tropical conditions, roughly 20°C (peaks in summer up to 33°C) and 70% relative humidity (RH) day and night, with natural light during winter and summer.

After acclimation, plants were moved into a climate chamber under a 12‐h photoperiod. Day/night conditions were established in increments (Table S2), with day set to maximum 35°C, 200 μmol m^−2^ s^−1^, 60% RH, and night, minimum 15°C, 70% RH. Although abundant in the greenhouse, little to no prey were present in the climate chambers.

Total water withholding was applied in the climate chambers to create stressful conditions and test for facultative CAM induction. However, *P. moranensis* and *P. emarginata* showed excessive stress and therefore received ca. 100 mL water once per week. Since *P. grandiflora* is originally from temperate conditions and was only acquired in time for inclusion in the summer trial, this species was acclimated directly in the climate chamber (Plant Growth Chamber E‐22 L, Percival Scientific, Perry, Iowa, USA) at 200 μmol m^−2^ s^−1^, 15°C day and 8°C night (12‐h light period), at constant 80% RH.

### Sample collection for winter and summer trials

Although several species exhibit seasonal heterophylly (Table 1), summer (wet season) and/or winter (dry season) leaves were sometimes present, probably because plants were kept in an artificial environment. As such, young to adult and healthy‐looking leaves were sampled for subsequent analyses, representing the leaf type matching the season of the trial. Individuals from the winter trial were reused in the summer trial whenever possible (Tables S3 and S4). Because of the extremely small size of some species, when there was not enough plant material for three replicates for titratable acidity, values were marked as unavailable (NA), and the group was not included in further statistical analyses or in the boxplots.

The following sampling routine was used both for winter and summer trials. Samples were collected once in the greenhouse (well‐watered (WW) plants) and subsequently three times, once per week, as water withholding intensified after being moved into the climate chamber (time points Week 1–Week 3). Leaf samples were collected at dusk (16:30 h) and dawn (06:50 h) from the same plants. This corresponds roughly to 2.5 h before lights were switched off for dusk and immediately before switching lights on for dawn in the climate chambers. Whole leaves were harvested and immediately frozen in liquid nitrogen. All samples were stored at −20°C until use.

### Titratable acidity

Sample processing followed Siadjeu & Kadereit (2024). Briefly, ca. 10–60 mg leaf material was weighed and incubated at 60°C in 3 mL 20% ethanol for 60 min. The extract was divided into three technical replicates of 1 mL and neutralized by adding 0.002 M NaOH in increments of 5 μL or less, with bromothymol blue as indicator. The measured values were later normalized to fresh weight (FW) and converted to μmol H^+^ g^−1^ FW. Differences in acidity levels between dawn and dusk (ΔH^+^) were calculated by subtracting values of corresponding samples.

### Leaf anatomy of *P. esseriana*

For assessment of mesophyll and bundle‐sheath anatomy, fresh summer leaf material was collected from *P. esseriana* ‘El Huizache’ as representative species, and embedded in 4% agarose (Agarose Standard, no. 3810.2; Carl Roth GmbH + Co. KG, Karlsruhe, Baden‐Württemberg, Germany) in 1x PBS buffer (obtained by diluting in ddH_2_O: ROTI^®^Stock 10× PBS, article number 1058.1, Roth, Germany). Leaf cross‐sections of 90 μm thickness were then produced using a vibratome (Microm HM 650 V, Microm International, Thermo Fisher Scientific, Waltham, Massachusetts, USA). The unstained samples were observed and photographed under a standard brightfield microscope with an external camera (Sony α 6000 mounted with Sony SELP1650; Sony Europe, Weybridge, UK) in 1x PBS buffer as slide medium. The resulting images were manually adjusted for contrast and brightness in Edit (Sony Corporation, v. 3.6.00.01200).

### Gas exchange monitoring of *P. agnata*

Using an entire droughted leaf rosette of *P. agnata* (no water for 33 days), gas exchange measurements were performed using a Walz gas‐exchange and fluorescence system GFS‐3000 with a standard cuvette and LED array (Walz, Effeltrich, Germany). *Pinguicula agnata* was chosen as study system because: (1) it has larger leaves compared to the other species and the entire rosette could be enclosed in the cuvette, (2) both summer and winter leaves are succulent, and (3) it had consistently high ΔH^+^ in the acid test. The rosette with fully expanded summer leaves was excised from the pot and roots were mechanically cleaned of soil particles. No visible hibernaculum was present at the time of measurement. The external CO_2_ concentration was 400 ppm inside the cuvette throughout the experiment. The photon flux density (PPFD) was 400 μmol m^−2^ s^−1^ during the day, reduced to 200 μmol m^−2^ s^−1^ for 2 h in the morning (dawn) and in the evening (dusk), with a light period of 11 h (and dark period of 13 h). Temperature inside the cuvette followed ambient temperatures. *A*, *E* and *g*_*s*_ were stored every 10 min throughout the measurement period, with short breaks in order to zero the IRGA once per hour. At the end of the experiment, all leaves of the rosette were separated and scanned on scale paper to determine leaf area which was used to normalize the data.

### Statistical tests

All analyses and plots were assessed in R (v. 4.4.2 (2024‐10‐31 ucrt)) implemented in RStudio (v. 2024.12.0). Values of ΔH^+^ were compared between watering conditions in each species using pairwise comparisons (two‐sample *t*‐test), and later between different species in each watering condition. For the latter (species comparison), statistical tests including more than two means were chosen after checking for normality (Shapiro–Wilk test) and variance (Levene test), normal and equal variances (ANOVA + Tukey test), normal and unequal variances (Welch ANOVA + Games Howell), non‐normal and equal variances (Kruskall‐Wallis + Dunn's test), and non‐normal and unequal variance were transformed and fitted to the other conditions.

## RESULTS

In total, eight *Pinguicula* species (nine counting the two accessions of *P. esseriana*) were studied in the present work across one winter and one summer trial (Table 1 and Table S1): *P. agnata, P. emarginata. P. esseriana* ‘El Huizache’, *P. esseriana* ‘Ciudad Victoria’, *P. gigantea, P. grandiflora, P. laxifolia, P. martinezii*, and *P. moranensis*. All species occur in Mexico in subtropical conditions except *P. grandiflora*, which is found in Europe in temperate climates (Table 1). In terms of leaf structure, four species (i.e. *P. agnata, P. esseriana* ‘El Huizache’, *P. esseriana* ‘Ciudad Victoria’, *P. laxifolia*, and *P. moranensis*) were previously described to present heterophylly from winter to summer: winter leaves are non‐carnivorous and succulent, summer leaves are carnivorous (Table 1, Fig. 1). The remaining three Mexican species are homophyllous: *P. emarginata. P. gigantea*, and *P. martinezii*. In addition, the temperate heterophyllous, perennially wet‐growing European species *P. grandiflora* was used for comparison in the summer trial.

### Titratable acidity in leaves of *Pinguicula* revealed significant circadian differences, but not for all species

**Fig. 2:**
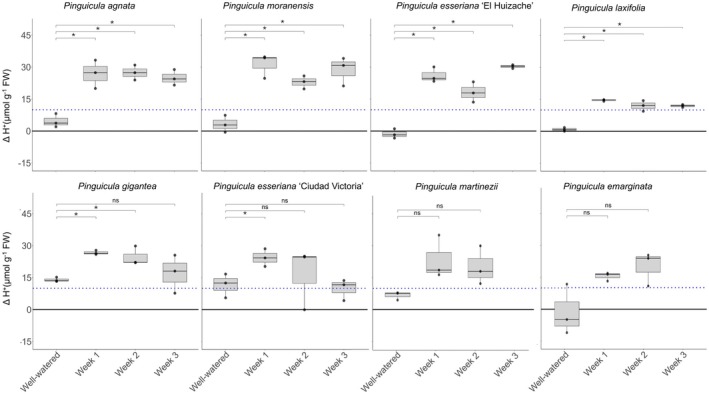
Titratable acidity measured during the winter trial for seven species of *Pinguicula*, *P. agnata, P. moranensis, P. esseriana* (two different accessions), *P. laxifolia, P. gigantea, P. martinezii* and *P. emarginata*. Plants were acclimated in greenhouse conditions for 2 weeks and kept well‐watered (well‐watered treatment), subsequently transferred to climate chambers and subjected to 1, 2 or 3 weeks of water withholding (Weeks 1, 2 and 3). See Methods for more details. ΔH^+^ was calculated subtracting samples at dawn from samples at dusk. Blue‐dotted line is set at 10 μmol H^+^ g^−1^ FW as a threshold for a meaningful variation in circadian titratable acidity. Boxplots show data (n = 3, dots) and pairwise comparisons (two‐sample *t*‐test) between well‐watered and water withheld treatments for each species. Asterisks indicate *P* > 0.05 and “ns” is non‐significant comparisons. Raw values are presented in Table S3. Unfortunately, less than three replicates were collected for *P. emarginata* and *P. martinezii*, hence data were not plotted but are included in Table S3.

The winter trial included *P. agnata, P. emarginata, P. esseriana* ‘El Huizache’ in *P. esseriana* ‘Ciudad Victoria’, *P. gigantea, P. laxifolia, P. martinezii* and *P. moranensis*. In this trial, most species had ΔH^+^ values <10 μmol H^+^ g^−1^ FW under well‐watered (WW) conditions, except for *P. gigantea* (Fig. 2). Even after 1 week without irrigation, *P. agnata, P. moranensis, P. esseriana* ‘El Huizache, and *P. laxifolia* showed a significant increase in ΔH^+^, and subsequent values for Weeks 2–3 were similar to those of Week 1 (Fig. 2). In the remaining species, there was a significant difference in ΔH^+^ for WW in Weeks 1–2 in *P. gigantea*, WW only in Week 1 in *P. esseriana* ‘Ciudad Victoria’, and only a trend of increasing ΔH^+^ from WW to Weeks 1–2 in *P. martinezii* and *P. emarginata* (Fig. 2). When comparing ΔH^+^ across *Pinguicula* species and considering distribution of the collected data, *P. gigantea* had the highest ΔH^+^ in WW, *P. laxifolia* had the lowest ΔH^+^ (Fig. S1), and *P. agnata* had the most consistent induction throughout the winter experiment (Fig. 2). The two replicates that could be sampled for *P. martinezii* in Week 3 showed a similar trend to Week 2, but, unfortunately, there was insufficient material of *P. emarginata* for sampling in Week 3 (Table S3).

**Fig. 3:**
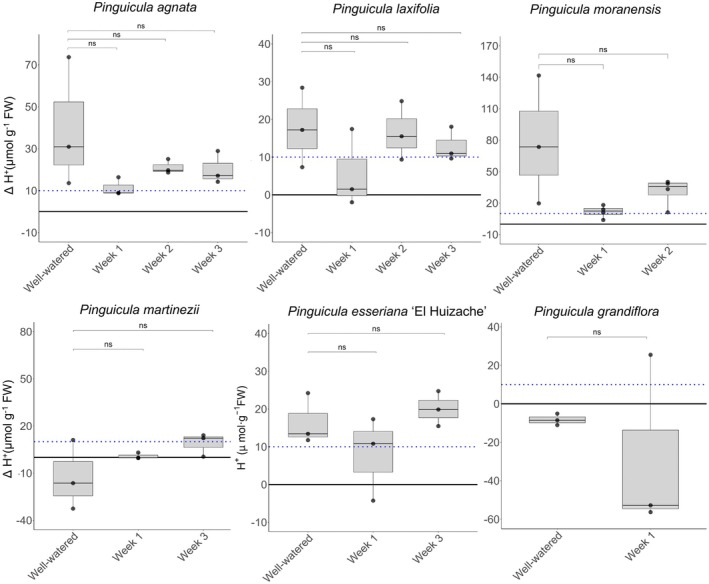
Titratable acidity measured during the summer trial for five species of *Pinguicula*, namely *P. agnata, P. laxifolia, P. moranensis, P. martinezii, P. esseriana*, and *P. grandiflora*. The species list from the winter to summer trial changed since some did not survive or had very limited material. Plants were kept in a greenhouse after winter and well‐watered (well‐watered treatment), subsequently transferred to climate chambers and subjected to 1, 2 or 3 weeks of water withholding (Weeks 1, 2 and 3). See Methods. ΔH^+^ was calculated by subtracting samples taken at dawn from samples taken at dusk. Blue‐dotted line is set at 10 μmol H^+^ g^−1^ FW as a threshold for a biologically meaningful variation in circadian titratable acidity. Boxplots show data (n = 3, indicated by dots) and pairwise comparisons (two‐sample *t*‐test) between well‐watered and water withholding treatments for each species. Asterisks indicate *P* > 0.05 and “ns” are non‐significant. Raw values are in Table S4. Data are available for *P. emarginata* at Week 2 in Table S4, together with less than three replicates for *P. martinezii*, and *P. esseriana* at Week 2.

For the summer trial, *P. agnata, P. esseriana* ‘El Huizache’, *P. laxifolia, P. martinezii*, and *P. moranensis* were retained, and *P. grandiflora* was added. Interestingly, there were no significant differences between WW and time points following 1, 2 or 3 weeks of water withholding treatment, and the data collected showed a scattered distribution (Fig. 3 and Table S4). For *P. agnata, P. laxifolia* and *P. moranensis*, which had clearly increased ΔH^+^ in the winter trial, there was no trend of increasing ΔH^+^ throughout the weeks without water. For *P. esseriana* this was also true in the summer trial, and for *P. martinezii* there was a very subtle increase (Fig. 3). The only species being tested for the first time and grown in the climate chamber since the start of the summer trial, *P. grandiflora*, did not show a significant increase in ΔH^+^, although one of the three replicates collected after 2 weeks without irrigation did have a positive ΔH^+^ of 20.09 μmol H^+^g^−1^ FW (Table S4).

### *Pinguicula* showed no evidence of C_4_ photosynthesis and reduced respiration in the dark

**Fig. 4:**
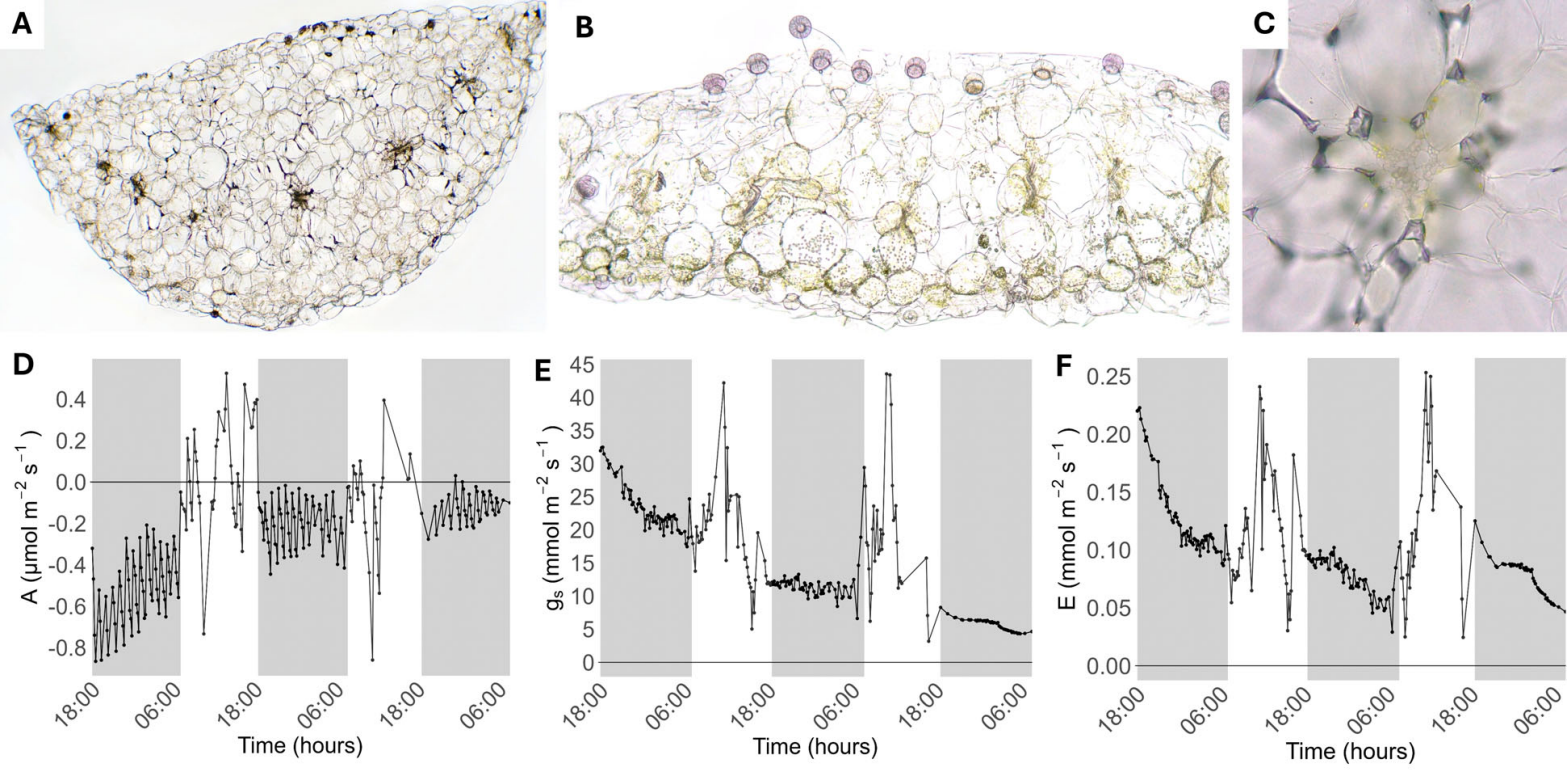
Leaf anatomy and gas exchange of representative *Pinguicula* species. (A–C) Cross‐sections of summer leaves from *P. esseriana*. A. Proximal cut (close to main axis of plant). B. Distal cut (closer to leaf edge) with glands clearly visible. C. Detail of vascular bundle without Kranz anatomy of C_4_ plants. A‐B. 200× magnification, C. 400×. (D–E) Gas exchange data for *P. agnata* collected for 3 days. D. Net assimilation (*A*). E. Stomatal conductance (*g*_*s*_). F. Transpiration (*E*). Shaded areas represent dark time.

The cross‐sections of summer leaves of *P. esseriana* revealed a single layer of epidermal cells on both leaf surfaces, with glands on the adaxial surface of the basal region (Fig. 4). In the mesophyll, there were prominent, large water storage (hydrenchyma) cells of irregular size. Chlorenchyma and hydrenchyma cells did not differ in size, resulting in homogenous mesophyll. The vascular bundles were centrally distributed with no conspicuous Kranz anatomy (Fig. 4). Overall, the leaves were very fragile and soft, making it challenging to obtain good sections.

*Pinguicula agnata* was chosen as representative, heterophyllous subtropical species and subjected to 33 days of water withholding. Our goal was to provide prolonged stress that would induce a transition to dark CO_2_ assimilation. Gas exchange at the end of this period showed a negative *A* at night, with more negative values immediately after the end of light, becoming less negative until the end of the dark period, especially on the third monitored night (Fig. 4). During the day, *A* was low and decreased even further with increased duration of water withholding, with similar responses of *g*_*s*_ and *E* (Fig. 4).

## DISCUSSION

In the current work, we assessed the induction of CAM in seven Mexican and one European species of the carnivorous genus, *Pinguicula*, cultivated under controlled conditions following 1–3 weeks of withholding water. Our list of *Pinguicula* tested for CAM included species from more humid (*P. grandiflora*, *P. emarginata*, *P. martinezii*) (Table 1; Lampard *et al*. 2016, Roccia *et al*. 2016) or more arid environments (*P. agnata, P. esseriana*) (Table 1; Kirchner 1981; Lampard *et al*. 2016), as well as heterophyllous (*P. agnata, P. moranensis, P. esseriana, P. laxifolia*) and homophyllous (*P. gigantea, P. martinezii, P. emarginata*) representatives. The leaf anatomy study performed here in *P. esseriana* confirmed the previously described organization of the mesophyll (Stanescu and Toma 2007), but discarded the earlier report of C_4_ photosynthesis in *Pinguicula* (Studnička 1991), based on the lack of a conspicuous Kranz anatomy. In addition, leaf anatomy of homophyllous *P. gigantea* also had no evidence of Kranz anatomy (Reut *et al*. 2021). Nevertheless, follow‐up studies in the group should consider seasonal differences between winter and summer leaves, since a comparison was not included in the present work.

Overall, ΔH^+^ values above 10 μmol H^+^ g^−1^ FW and significantly higher than under WW conditions were considered as evidence of CAM induction. During the winter trial and following the absence of water, five species (i.e. *P. agnata, P. moranensis, P. laxifolia, P. gigantea, P. esseriana* ‘El Huizache’ and *P. esseriana* ‘Ciudad Victoria’) were successfully confirmed as performing CAM. The accumulated H^+^ was in the range 15–30 μmol H^+^ g^−1^ FW for these species, which matches literature reports for other facultative and weak CAM species (Winter 2019; Winter & Smith 2021). In addition, the slightly less negative values of *A* from the start to the end of the dark period measured here in *P. agnata* could indicate weak CAM, due to recycling of nocturnal CO_2_ from respiration (Winter & Holtum 2014; Winter 2019). Among these five species, all are heterophyllous, with succulent, non‐carnivorous winter leaves, except *P. gigantea*, which is homophyllous, that is, carnivorous leaves formed throughout winter and summer but reportedly very succulent (Luhrs 1995).

Interestingly, no other homophyllous species tested showed a clear pattern of CAM induction. *Pinguicula martinezii* and *P. emarginata* showed a trend to increased ΔH^+^, but the differences between time points were not significant (Fig. 2). Similarly, there was no significant increase in ΔH^+^ following water withholding in the European *P. grandiflora* (Fig. 3). The lack of CAM activity is potentially consistent with the mesophytic to wet conditions in the habitats of these species (see Table 1 and references therein). However, considering experimental difficulties and scarcity of plant material after several rounds of sampling, CAM still cannot be completely ruled out in these species.

Our sampling was not sufficient to correlate climate of the place of origin of the *Pinguicula* spp. with CAM expression and draw significant conclusions, although previous studies have reported differences in habitat precipitation levels of the species studied here (Table 1). A lack of correlation between CAM intensity and climate of the place of origin was also reported for *Portulaca oleracea* (Portulacaceae) (Ferrari *et al*. 2020). Interestingly, the two accessions of *P. esseriana* tested in the present study performed slightly differently regarding CAM expression and therefore might suggest intraspecific variation in CAM expression, even after long cultivation in the same greenhouse (Fig. 2). Plants from the species type locality, a high plateau of ca. 2850 m a.s.l. near El Huizache, San Luis Potosí state, were collected in 1977 by G. Köhres from Bochum Botanical Garden and since maintained in cultivation in botanical gardens (Kirchner 1981). *Pinguicula esseriana* ‘Ciudad Victoria’ originates from 1450 m a.s.l. near Ciudad Victoria, Tamaulipas state, collected in 1990 by P. Debbert from Munich and since maintained in cultivation in the Botanical Garden, often under the name “*P. jaumavensis*”. The relationship between climate and CAM expression in *Pinguicula* remains an open topic for further research.

In terms of the versatility in CAM expression in *Pinguicula*, when the same individuals were used from the winter to the summer trial, ΔH^+^ remained high, with no significant differences between WW and water‐deprived plants (Fig. 3). Furthermore, there was no proportional intensification of CAM in the form of increasing ΔH^+^ during soil drying in Weeks 2–3 compared to Week 1 (Figs. 2, 3). This is an interesting feature to understand the adaptive significance of facultative and weak CAM in carnivorous plants, with CAM possibly being a survival strategy during drier months, instead of a metabolic response that builds with increasing stress. This weak CAM induction might: (1) supply CO_2_ throughout the day to maintain photosynthesis and sustain electron flow from light reactions, avoiding CO_2_ starvation related to reduced g_s_ during drought; (2) increase water use efficiency, and (3) prevent photoinhibition (Herrera 2009), but possibly not sustaining growth for extended periods. The overall contribution of weak CAM to reproductive success or survival rate of individual plants in *Pinguicula* remains an open question. In general, the adaptive significance of weak CAM is a topic that awaits further exploration.

The interplay between N and C metabolism deserves attention in future CAM studies with *Pinguicula* species. A study in the epiphyte *Guzmania monostachia* (Bromeliaceae), which also grows in nutrient‐poor environments, showed that N deficiency resulted in increased CAM expression (Rodrigues *et al*. 2014). Nonetheless, in *Kalanchoë* (Crassulaceae) and representative Cactaceae, CAM expression is maximized at optimal N levels and reduced during N deficiency (reviewed in Pereira & Cushman 2019). This highlights a species‐specific relationship between N nutrition and CAM and suggests trade‐offs for water deficiency and nutrient deficiency in relation to CAM, which should be explored.

A sufficient nutrient supply, especially nitrogen (N), is essential to maximize *A*, and one strategy to survive in nutrient‐poor habitats is the evolution of carnivory (e.g. Givnish *et al*. 1984, 2018; Fleischmann *et al*. 2018; Pavlovič 2022). Méndez & Karlsson (1999) reported *A* of 2–3 μmol CO_2_ m^2^ s^−1^ in non‐succulent *P. vulgaris, P. alpina*, and *P. villosa* from Sweden. In *Drosera rotundifolia*, *D. capensis* and *Sarracenia leucophylla*, *A* of 1.32–2.22 μmol CO_2_ m^2^ s^−1^ was also reported (Bruzzese *et al*. 2010). Low rates of *A* in carnivorous plants could be linked to the high cost of the biochemical, anatomical, and ultrastructural adaptations of their traps. Corresponding leaf modifications necessary for carnivory (bright colouration, secretions, morphology for prey attraction and retention) usually result in smaller leaves and/or leaf morphologies less suitable for effective photosynthesis (Pavlovič 2022). Hence, a low *A* could be a cause and a result of the slower growth rates of carnivorous plants, leading to low competitive ability compared to non‐carnivorous species (Hájek & Adamec 2010). In fact, *A* in terrestrial carnivorous plants has been reported to be lower than in non‐carnivorous plants (Ellison & Gotelli 2001; Hájek & Adamec 2010; Pavlovič 2022). Nevertheless, when comparing carnivorous plants and non‐carnivorous plants growing in the same nutrient‐poor environment, all plants had similar N, P, K, and δ^13^C (Givnish and Shiba 2022). Although there were no photosynthesis measurements in that study, N, P and K levels suggest that *A* might be similarly limited in carnivorous and non‐carnivorous plants and highlights the need to compare the two in the same environment.

The general lack of drought adaptations in carnivorous plants is unsurprising, given the strong association of the carnivorous syndrome with wet habitats (Givnish *et al*. 2018). Nevertheless, as in *Pinguicula*, succulence is also found in a few carnivorous plants within Lentibulariaceae (Lamiales). *Genlisea uncinata* and *G. oligophylla* form succulent photosynthetic inflorescence scapes (Taylor 1989; Fleischmann 2012; Płachno *et al*. 2020). Although CAM has been detected in other Lamiales (Gilman *et al*. 2023), it has not been detected in any Lentibulariaceae or close relatives. Thus, the *Pinguicula* CAM lineage seems to be of independent origin from the other known Lamiales CAM lineages, for example, *Littorella* (Plantaginaceae), *Haberlea* + *Ramonda*, *Codonanthopsis* (Gesneriaceae), *Marrubium* and *Coleus* (Lamiaceae). Further research should include broader sampling of succulent carnivorous plants in Lentibulariaceae to assess facultative CAM. Moreover, especially considering reports on CAM in combination with C_4_ photosynthesis in *Portulaca oleracea* (Koch & Kennedy 1980) and *Sesuvium sesuvioides* (Siadjeu & Kadereit 2024), a new CAM screening could include carnivorous species from Caryophyllales. Overall, our current findings underline once more the multitude and complexity of CAM physiotypes and reiterate the importance of further describing and understanding CAM diversity.

## AUTHOR CONTRIBUTIONS

AF, GK and TM designed the research, NJF performed the experiments, NJF, RCF and TM analysed the data. NJF generated the first version of the manuscript. All authors interpreted the results and contributed to manuscript writing.

## CONFLICT OF INTEREST

The authors declare that there is no conflict of interests.

## Supporting information


**Table S1.** Accession numbers of the Botanical Garden Munich, Germany.
**Table S2.** Settings for temperature, relative humidity (RH), CO_2_ concentration, and light intensity in the climate chamber.
**Table S3.** Titratable acidity measured during the winter trial for seven species of Pinguicula.
**Table S4.** Titratable acidity measured during the summer trial for seven species of Pinguicula.

**Fig. S1.** Titratable acidity measured during the winter trial for *Pinguicula*. Plants were acclimated in a greenhouse for 2 weeks and well‐watered (well‐watered treatment), subsequently transferred to climate chambers and subjected to 1, 2 or 3 weeks of water withholding (Weeks 1, 2 and 3). See Methods. ΔH^+^ was calculated by subtracting samples taken at dawn from samples taken at dusk. Blue‐dotted line is set at 10 μmol H^+^ g^−1^ FW as a threshold for a meaningful variation in circadian titratable acidity. Boxplots show data (n = 3, indicated by dots) and different letters indicate significant differences (*P* > 0.05) when comparing means for all species for a given sampling point: (A) well‐watered conditions when plants were kept in the greenhouse, (B) One week following the start of water withholding treatment, (C) Two weeks following the start of water withholding treatment. (D) Three weeks following the start of water withholding treatment. Raw values are presented in Table S2.
**Fig. S2.** Titratable acidity measured during the summer trial for species of *Pinguicula*. The species list from winter to summer changed since a few species did not survive or had very limited material. Plants were kept in a greenhouse after the winter trial and well‐watered (well‐watered treatment), subsequently transferred to climate chambers and subjected to 1, 2 or 3 weeks of water withholding (Weeks 1, 2 and 3). See Methods section. ΔH^+^ was calculated by subtracting samples at dawn from samples at dusk. Blue‐dotted line set at 10 μmol H^+^ g^−1^ FW as threshold for a biologically meaningful variation in titratable acidity. Boxplots show data (n = 3, indicated by dots) and different letters indicate significant differences (*P* > 0.05) when comparing means for all species for a given sampling point: (A) well‐watered conditions with plants kept in the greenhouse, (B) One week following start of water withholding, (C) Two weeks following the start of water withholding. (D) Three weeks following start of water withholding. Raw values are presented in Table S3.
