# Supplementary material for: Yes, we CAM! First evidence of CAM photosynthesis in a carnivorous plant
Source: Plant Biol (Stuttg). 2025 Nov 10;28(1):272–81. doi: 10.1111/plb.70128 (PMC12710813; doi:10.1111/plb.70128)
Supplement: Supplementary file 2 — Fig. S1. Titratable acidity measured during the winter trial for Pinguicula. Plants were acclimated in a greenhouse for 2 weeks and well‐watered (well‐watered treatment), subsequently transferred to climate chambers and subjected to 1, 2 or 3 weeks of water withholding (Weeks 1, 2 and 3). See Methods. ΔH+ was calculated by subtracting samples taken at dawn from samples taken at dusk. Blue‐dotted line is set at 10 μmol H+ g−1 FW as a threshold for a meaningful variation in circadian titratable acidity. Boxplots show data (n = 3, indicated by dots) and different letters indicate significant differences (P > 0.05) when comparing means for all species for a given sampling point: (A) well‐watered conditions when plants were kept in the greenhouse, (B) One week following the start of water withholding treatment, (C) Two weeks following the start of water withholding treatment. (D) Three weeks following the start of water withholding treatment. Raw values are presented in Table S2. Fig. S2. Titratable acidity measured during the summer trial for species of Pinguicula. The species list from winter to summer changed since a few species did not survive or had very limited material. Plants were kept in a greenhouse after the winter trial and well‐watered (well‐watered treatment), subsequently transferred to climate chambers and subjected to 1, 2 or 3 weeks of water withholding (Weeks 1, 2 and 3). See Methods section. ΔH+ was calculated by subtracting samples at dawn from samples at dusk. Blue‐dotted line set at 10 μmol H+ g−1 FW as threshold for a biologically meaningful variation in titratable acidity. Boxplots show data (n = 3, indicated by dots) and different letters indicate significant differences (P > 0.05) when comparing means for all species for a given sampling point: (A) well‐watered conditions with plants kept in the greenhouse, (B) One week following start of water withholding, (C) Two weeks following the start of water withholding. (D) Three weeks following start [file PLB-28-272-s002.docx]

Supplementary material

Title: Yes, we CAM! First evidence of CAM photosynthesis in a carnivorous plant

Authors: N. Joris Fleck, Thibaud F. E. Messerschmid, Andreas Fleischmann, Renata Callegari Ferrari, Gudrun Kadereit

List:

| Supplementary Figures |
| --- |
| Fig S1 Titratable acidity measured during the winter trial for species of *Pinguicula* – comparing species statistically |
| Fig. S2 Titratable acidity measured during the summer trial for species of *Pinguicula* – comparing species statistically |

| Supplementary Tables (Excel file) |
| --- |
| Supplementary Table S1. Accession numbers of the Botanical Garden Munich, Germany |
| Supplementary Table S2. Settings of temperature, relative humidity (RH), CO_2_ concentration, and light intensity in the climate chamber |
| Supplementary Table S3. Titratable acidity measured during the winter trial for seven species of Pinguicula |
| Supplementary Table S4. Titratable acidity measured during the summer trial for seven species of Pinguicula |


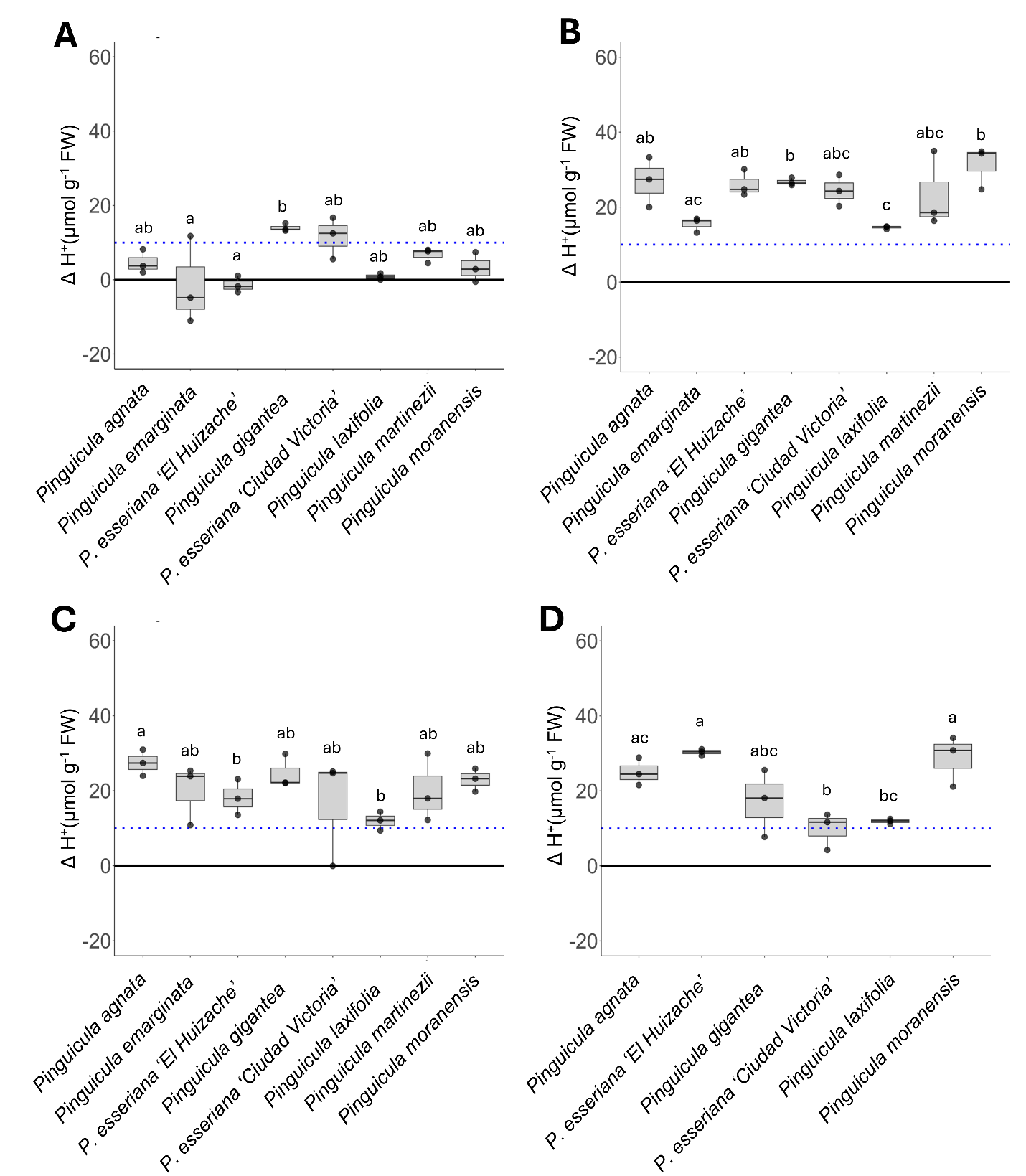


Fig. S1. Titratable acidity measured during the winter trial for species of *Pinguicula.* Plants were acclimated in greenhouse conditions for 2 weeks and kept well-watered (well-watered treatment), subsequently transferred to climate chambers and subjected to 1, 2 or 3 weeks of water withholding treatment (weeks 1, 2 and 3). More details are given in the methods section. ΔH^+^ was calculated subtracting samples taken at dawn from samples taken at dusk. The blue-dotted line is set at 10µmol H^+^ g^-1^ FW as a threshold for a biologically meaningful variation in circadian titratable acidity as discussed in the literature. Boxplots show data (n = 3, indicated by dots) and different letters indicate significant differences (p>0.05) when comparing means for all species for a given sampling point: **A** well-watered conditions when the plants were kept in the greenhouse, **B** One week following the start of the water withholding treatment, **C** Two weeks following the start of the water withholding treatment. **D** Three weeks following the start of the water withholding treatment. Raw values are presented in Supplementary Table S2.


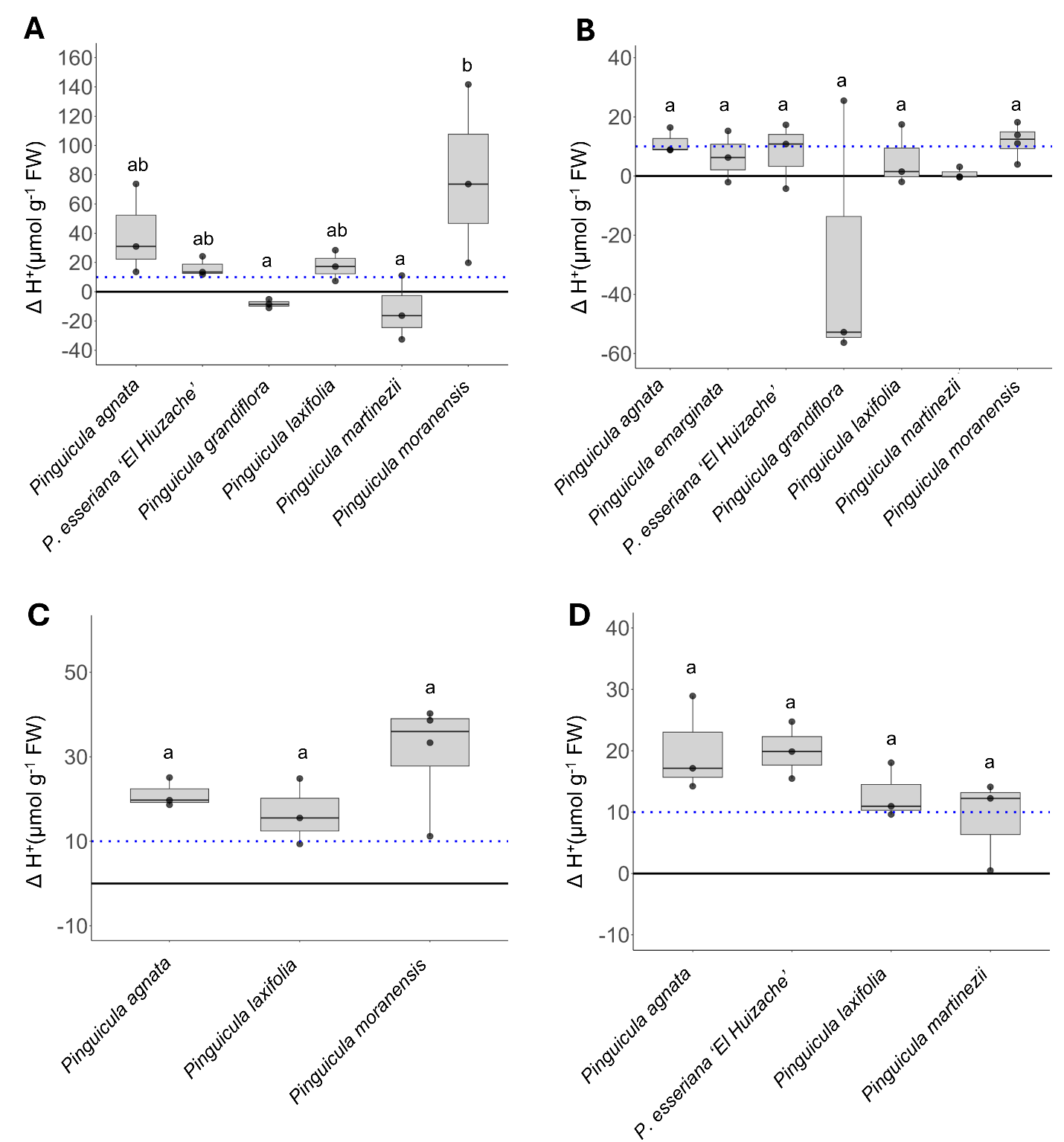


Fig. S2. Titratable acidity measured during the summer trial for species of *Pinguicula.* The species list from winter to summer trial changed since a few species did not survive or presented very limited amount of material. Plants were kept in greenhouse conditions after the winter trial and kept well-watered (well-watered treatment), subsequently transferred to climate chambers and subjected to 1, 2 or 3 weeks of water withholding treatment (weeks 1, 2 and 3). More details are given in the methods section. ΔH^+^ was calculated subtracting samples taken at dawn from samples taken at dusk. The blue-dotted line is set at 10µmol H^+^ g^-1^ FW as a threshold for a biologically meaningful variation in circadian titratable acidity as discussed in the literature. Boxplots show data (n = 3, indicated by dots) and different letters indicate significant differences (p>0.05) when comparing means for all species for a given sampling point: **A** well-watered conditions when the plants were kept in the greenhouse, **B** One week following the start of the water withholding treatment, **C** Two weeks following the start of the water withholding treatment. **D** Three weeks following the start of the water withholding treatment. Raw values are presented in Supplementary Table S3.
